# Ipecacuan—Asthma

**Published:** 1844-08

**Authors:** 


### IPECACUAN-ASTHMA.

For a report of the following interesting case we are indebted to a student of medicine in the Medical Institute of Louisville, Mr. S. T. Payne, of Tennessee. The particulars of it will recall to the memory of the reader a similar case reported by Dr. Felix Robertson, of Nashville, in the 8th volume of this Journal. The multiplication of such cases will put practitioners and apothecaries on their guard against unnecessarily exposing their lungs to the noxious influence of ipecacuanha. Mr. Payne says:

"In filling a jar with ipecac., one day last spring, I incautiously inhaled a portion of it with the air while holding my head over the dust that rose. Very soon I was affected with a peculiar uneasiness in the chest which caused me to withdraw. This becoming somewhat less, I returned to the operation, but before I had emptied all the article out of the paper the sensation in the lungs grew so distressing that I became alarmed. The symptoms of my case at this moment were similar to those described by practical authors as characteristic of asthma. I had great difficulty of breathing, with a sense of suffocation, a dry, hacking cough, effusion of tears, quick pulse, violent headache, &c. The first thing I did was to take an emetic of ipecac., but I derived no benefit from it. Various minor remedies were tried, but I got no relief till I had blood drawn from my arm, which was done on the third day, when all the symptoms indicated the access of inflammation. I found it necessary to resort three times to venesection, and it was only after the third bleeding that the dyspnœa and other distressing symptoms gave way. Subsequently, by the advice of a physician who was called in, and who pronounced my case a dangerous one, I used with advantage various anti-spasmodics, lobelia, and the warm bath, and finally recovered in three weeks. Soon after getting about, I had occasion to administer an emetic of ipecac. to a patient, and although I handled the medicine cautiously, to my astonishment and great alarm, I was attacked with the symptoms from which I had just been suffering so severely. They were much milder, however, and continued for a shorter time. I find it now impracticable to handle ipecac. at all without experiencing unpleasant sensations in my chest. At the time that I was attacked I had not read the report of Dr. Robertson's case, and had no suspicion, for some time, that my illness was brought on by inhaling the ipecac."

Watson, in his Lectures, (p. 643) alludes to cases of this description, and proposes for the affection the name which we have adopted. It appears to be precisely analogous to hay-asthma, of which English physicians have written under the name of 'summer cold,' or 'catarrhus æstivus,' as quoted by Bostock in the Medico-Chirurgical Transactions. Of this affection Elliotson in his Practice (p. 772, et seq.) relates a number of striking and interesting cases. The same writer mentions instances in which a similar train of symptoms was excited by ipecacuan. He says "I heard a physician say that there was a case related on which he could depend—though I would not myself vouch for its accuracy,—of a person who had such a susceptibility of ipecacuanha, that on entering a room and being seized with asthma, he declared that there was ipecacuanha about. It was at first denied; but at last some one recollected that there was a box of ipecacuanha-lozenges in a table drawer. That," he adds, "was going very far; but it is a fact that some persons are seized with asthma, if ipecacuanha be near them."

In Dr. Robertson's case it was once brought on, we believe, by the application of the tip of his tongue to a vial-cork moistened with wine of ipecac. We know a highly respectable and intelligent lady in this neighborhood, who has had repeated attacks of an affection resembling asthma from slight exposure to the dust of ipecacuan. She is seized with these symptoms, we have been assured, if a dose of the medicine be dissolved in the room where she is.

The kindred affection—hay-asthma—is supposed to be occasioned by the pollen of flowers. Persons troubled with it derive relief by going to the sea-shore, beyond the reach of flowering plants. They are most painfully affected by walking over meadows when the grass is in bloom. It is known that many persons are attacked by distressing symptoms while sleeping in apartments where there are flowers. We knew a lady who suffered exceedingly with dyspnœa during all the season while flowers were blooming around her house. Is it not probable that in these cases too it is the pollen which gives rise to the bronchial irritation?

The affection, whether excited by ipecacuan or flowering plants, does not seem to be asthma merely, but a combination of catarrh and asthma. It consists in excessive irritation of the mucous membrane of the eyes, nose, and the whole of the air passages. Elliotson, in view of the fact that the irritating substances are compound, proposes chlorine as a remedy for the complaint; and in several cases where it was tried, he states that the result was satisfactory. The patients inhaled the fumes from saucers filled with a solution of the chloride of lime or of soda. Y.

